# Toxicological Implications of Platinum Nanoparticle Exposure: Stimulation of Intracellular Stress, Inflammatory Response, and Akt Signaling In Vitro

**DOI:** 10.1155/2018/1367801

**Published:** 2018-10-01

**Authors:** Claudia J. Labrador-Rached, Rebecca T. Browning, Laura K. Braydich-Stolle, Kristen K. Comfort

**Affiliations:** ^1^Department of Chemical and Materials Engineering, University of Dayton, Dayton, OH 45469, USA; ^2^Molecular Bioeffects Branch, Bioeffects Division, Airmen Systems Directorate, Wright-Patterson Air Force Base, OH 45433, USA; ^3^Integrative Science and Engineering Center, University of Dayton, Dayton, OH 45469, USA

## Abstract

Due to their distinctive physicochemical properties, platinum nanoparticles (PtNPs) have emerged as a material of interest for a number of biomedical therapeutics. However, in some instances NP exposure has been correlated to health and safety concerns, including cytotoxicity, activation of cellular stress, and modification to normal cell functionality. As PtNPs have induced differential cellular responses* in vitro*, the goal of this study was to further characterize the behavior and toxicological potential of PtNPs within a HepG2 liver model. This study identified that a high PtNP dosage induced HepG2 cytotoxicity. However, lower, subtoxic PtNP concentrations were able to elicit multiple stress responses, secretion of proinflammatory cytokines, and modulation of insulin-like growth factor-1 dependent signal transduction. Taken together, this work suggests that PtNPs would not be overtly toxic for acute exposures, but sustained cellular interactions might produce long term health consequences.

## 1. Introduction

In recent years, nanoparticles (NPs) have emerged as a major research thrust, with extensive resources and efforts focused on generating and characterizing a library of unique nano-sized materials. Due to their enhanced surface area to volume ratio, NPs display differential behavior from their bulk counterparts, such as augmented catalytic potential, distinctive plasmonic signatures, and enhanced transport capabilities [1]. The unique physicochemical properties and behaviors associated with NPs have led to their incorporation into hundreds of consumer, medical, and industrial applications [2].

This surge in NP usage is accompanied by a corresponding rise in human exposure to these novel materials. The field of nanotoxicology was founded to explore the safety of NPs following introduction within biological systems [3]. Years of investigations have revealed the mounting challenge facing this field, as observed bioresponses are dependent upon unique, NP-specific physicochemical properties, including size, surface moiety, core composition, and morphology [4]. Documented nanotoxicological responses vary widely and can include cellular death, activation of numerous stress responses, genotoxicity, and developmental abnormalities* in vivo* [5, 6]. Beyond traditional toxicological events, NPs have been shown to modify basal cell functionality, even in the absence of cytotoxicity, including the activation of inflammatory and immune responses, modification to gene transcription patterns, and modulation of signal transduction pathways following external stimulation [7, 8].

Many noble metals NPs, including platinum (PtNPs), have gained considerable attention due to their unique plasmonic and catalytic potentials, making them attractive for emerging nano-based developments [9]. PtNPs, which were originally employed as a fuel catalyst additive, are now being researched as a core material for numerous biomedical applications. Potential therapeutics span the vast biomedical field and include diagnostic mediators, contrast agents for imaging, medical implants, drug delivery vehicles, and photothermal therapy compounds [10–12].

While other metal NPs, such as gold and silver, have undergone extensive nanotoxicological investigations, limited studies have thoroughly explored the safety of PtNPs. More importantly, there appears to be some discrepancy between published PtNP safety analyses. Previous works demonstrated either a biocompatibility or a great degree of cytotoxicity following PtNP exposure, depending on the cell model [13, 14]. Interestingly, PtNPs are well documented as reactive oxygen species (ROS) scavengers and have been shown to reduce intracellular reactive oxygen in the presence of other stressors [15, 16]. However, in some cases, PtNPs elicited negative bioresponses including the activation of cellular stress, as well as DNA damage and genotoxicity effects* in vitro* [17–19]. In a zebrafish model, PtNP addition resulted in developmental alterations and a concentration dependent drop in heart rate, demonstrating that PtNP-dependent effects translated into* in vivo* models [20]. The existence of conflicting reports regarding PtNP-induced reactions suggests that additional evaluations are required to better elucidate biological responses and ensure the safety of PtNP-derived applications.

The goal of this study was to enhance the current state of knowledge regarding cellular response following PtNP introduction* in vitro*. This work employed the human liver, HepG2, cell model as PtNPs have been shown to accumulate in the liver, making it a relevant nanotoxicological target [17]. Select endpoints included both traditional toxicological evaluations and biological responses frequently overlooked in safety evaluations, such as the activation of an inflammatory response and modulation of signal transduction pathways. This work demonstrated that PtNPs induced cytotoxicity and activated several stress pathways in a dose-dependent fashion. Moreover, PtNP exposure elicited a mild proinflammatory response and augmented phosphorylation of the critical signaling protein Akt, suggesting that chronic PtNP exposure could lead to long term health concerns.

## 2. Materials and Methods

### 2.1. Liver Cell Culture

HepG2, the human liver model utilized in this study, was purchased from American Type Culture Collection. The cells were maintained in tissue culture treated petri dishes and grown in RPMI-1640 medium supplemented with 10% fetal bovine serum (FBS) and 1% penicillin/streptomycin. The HepG2s were housed in a humidified incubator maintained at 37°C and 5% carbon dioxide.

### 2.2. Platinum Nanoparticle Characterization

The citrate coated, 70 nm PtNPs were purchased from nanoComposix in concentrated solution form. The PtNP stock was stored at 4°C in the dark to minimize modifications to physicochemical properties over time. Primary particle size and morphology were verified using transmission electron microscopy (TEM) on a Hitachi H-7600 microscope. The spectral signature of the PtNPs was visualized through ultraviolet visible (UV-Vis) spectroscopy on a Synergy 4 BioTek microplate reader.

For the remainder of PtNP characterization experiments, the particles were diluted to 25 *μ*g/mL in either water or media immediately before assessment. Extent of PtNP agglomeration was quantified via dynamic light scattering (DLS) on an Anton Paar Litesizer 500. The surface charge of the particles was determined via a zeta potential analysis, also carried out on the Anton Paar Litesizer. For ionic dissolution experimentation, the PtNP samples were incubated at 37°C for 24 hours, followed by NP removal via centrifugation at 10,000 rpm for 15 minutes. The ion containing supernatant was collected and analyzed for platinum content via inductively coupled plasma optical emission spectrometry (ICP-OES) on a Thermo Fisher iCAP 7200.

### 2.3. HepG2 Viability Assessment

HepG2 cells were seeded into 96-well plates at a density of 3x10^4^ cells per well and returned to the incubator. The following day the cells were washed and dosed with the denoted PtNP concentration or fresh media as a negative control. After a 24-hour exposure the cells were washed and the viability was determined using the CellTiter 96 AQueous One Solution Cell Proliferation Assay (MTS) from Promega, in accordance with the manufacturer's protocol. The untreated cells served as a control to determine percent viability of experimental conditions.

### 2.4. Intracellular Stress Evaluations

Stress levels within the HepG2 cells were assessed via two endpoints: reactive oxygen species (ROS) and actin levels. For both metrics HepG2 cells were seeded into black 96-well plates at a density of 3x10^4^ cells per well and returned to the incubator overnight. For ROS analysis, the cells were washed and incubated with DCFH-DA probe (Thermo Fisher Scientific), washed again, and exposed to the stated PtNP conditions for 24 hours.

For actin evaluation, the HepG2s were exposed to the denoted conditions for 24 hours, washed, and fixed with 4% paraformaldehyde. The cells were then probed for actin using Alexa Fluor 555-phalloidin (Thermo Fisher Scientific), in accordance with the manufacturer recommendations. After proper treatment and staining, both ROS and actin levels were quantified via fluorescence analysis on a Synergy 4 BioTek microplate reader. PtNP dosed conditions were normalized against an untreated, negative control.

### 2.5. HepG2 Inflammatory Response to PtNPs

Evaluation of proinflammatory cytokine production was used to assess activation of the HepG2 inflammatory response following PtNP exposure. Specifically this study examined the secretion of interleukin- (IL-) 1*β*, IL-6, IL-8, and tumor necrosis factor- (TNF-) *α*. For these evaluations, HepG2 cells were plated into 6-well plates at a density of 1x10^6^ cells per well and exposed to the stated conditions the following day. As FBS contains cytokines, PtNP-dosed and control experiments were carried out in serum free media. After an exposure duration of 24 hours the supernatants were collected and underwent cytokine analysis using protein specific ELISA kits from Thermo Fisher Scientific, in accordance with the manufacturer's directions.

### 2.6. Akt and Erk Signaling Activation

The liver cells were seeded at a density of 1x10^6^ cells per well and grown overnight in a 6-well plate. The cells were washed and dosed with the stated PtNP conditions in serum free media for a duration of 24 hours. The HepG2 cells were then stimulated with 10 ng/mL of insulin-like growth factor- (IGF-) 1 for 1 hour at 37°C in order to activate the PI3K/Akt and Ras/Erk pathways. The cells were then washed and lysed in a nondenaturing lysis buffer containing phosphatase and protease inhibitors (Cell Signaling Technology). The phosphorylation levels of Akt and Erk were determined using ELISA kits from Cell Signaling Technology, which targeted Ser473 and Thr202/Tyr204, respectively. Phosphorylation levels were normalized by the total amount of the same protein, determined via ELISA analysis (Cell Signaling Technology).

### 2.7. Statistical Analysis

All experiments were performed in three independent trials for the purpose of carrying out statistical analysis. All data is expressed as mean ± the standard error of the mean. Graphpad Prism was used to run a one-way ANOVA for statistical analysis, with a* p* value threshold set to 0.05, and an asterisk (*∗*) indicating significance from untreated controls.

## 3. Results and Discussion

### 3.1. PtNP Characterization

This study focused on evaluating the safety of PtNPs, as they have emerged as particles of interest spanning both the medical and commercial sectors [10–12, 21]. To date, however, conflicting reports exist regarding the safety of PtNPs within biological systems, thereby warranting additional evaluation. Prior to cellular exposure it was essential that the PtNPs underwent a standard battery of characterization assessments, as nanotoxicological potential has been correlated to the unique physicochemical properties of each experimental particle set [4].

**Figure 1:**
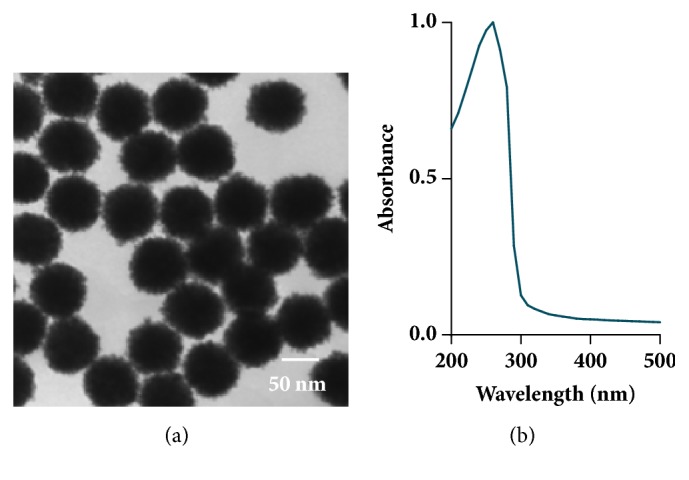
Characterization of the experimental PtNPs. (a) A representative TEM image of the experimental PtNPs was used to verify primary size and morphology. (b) The PtNP stock underwent UV-Vis analysis in order to obtain the unique spectral signature for these particles.

TEM imaging, shown in Figure 1(a), verified the spherical morphology of the 70 nm PtNPs utilized in this study. Using multiple images, a primary particle size of 68.3 ± 3.5 nm was calculated, demonstrating the uniformity of the PtNP stock. Next, the spectral profile of the PtNPs was produced using UV-Vis analysis (Figure 1(b)). This spectral signature displayed a single sharp peak, at approximately 260 nm, aligning with previous reports [22]. Moreover, the presence of a single peak confirmed the uniformity of the PtNPs, in agreement with TEM analysis.

Next, the PtNPs underwent characterization for behavioral trends following dispersion in water or cell culture media, thereby capturing both stock and exposure fluid conditions. As all nanomaterials will agglomerate to some degree in solution, it was necessary to verify that extensive agglomeration, and a loss of particle stability, did not occur. As shown in Table 1, the PtNPs displayed minimal agglomeration in water, with a slight increase noted within media, verifying particle stability. Moreover, the small polydispersion index (PdI) values indicated that the particle set was monodisperse. The increase in agglomerate size and PdI associated with cell culture media was due to the formation of a protein corona, which instantaneously forms around NP agglomerates within protein rich environments [23].

Zeta potential analysis was run to determine the surface charge of the particles (Table 1). In water the PtNPs displayed a negative charge, in accordance with the citrate coating. However, following dispersion in media, the PtNP surface charge was increased to approximately -10 mV, which aligns with the formation of the protein corona and the innate protein charge [23]. Finally, the percent of ionic dissolution was quantified over a 24-hour time period, to determine the rate of platinum ion production that occurred from the PtNP surface. Quantifying ion production has emerged as a critical characterization assessment, as the secretion of metallic ions has been correlated to cytotoxicity, in particular with silver NPs [24]. As seen in Table 1, the rate of ionic dissolution within water was relatively minimal and was further reduced following dispersion in media due to the presence of a protein coating.

### 3.2. HepG2 Viability and Stress Activation

**Figure 2:**
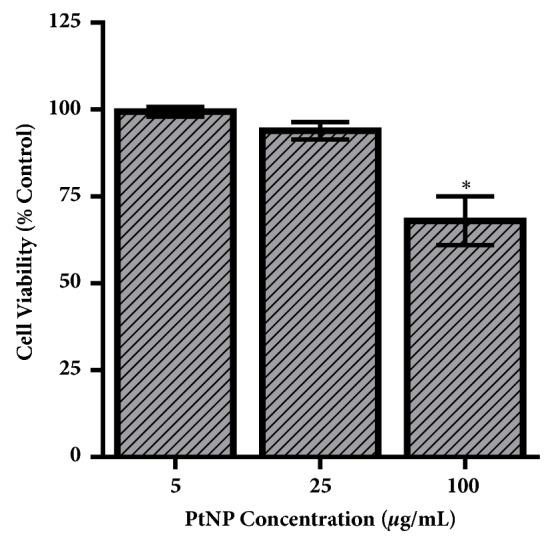
HepG2 viability following PtNP exposure. HepG2 liver cells underwent a 24-hour exposure to PtNPs at varying dosages, followed by evaluation of cellular viability. At the highest PtNP dosage, a significant toxicity response was observed. Data represents 3 independent trials with *∗* denoting statistical significance from untreated controls (*p* < 0.05).

Following PtNP characterization, a dose-dependent cytotoxicity analysis was carried out. HepG2 cells were specifically selected for this study owing to the considerations that PtNPs have been shown to accumulate in the liver and that this cell line has become a model for nanotoxicological investigations [13, 25]. As seen in Figure 2, PtNP exposure resulted in a dose-dependent decrease in HepG2 viability. At dosages of 25 *μ*g/mL or less, no toxicity was identified. However, at the high exposure concentration an approximate 25% cytotoxic response transpired.

**Figure 3:**
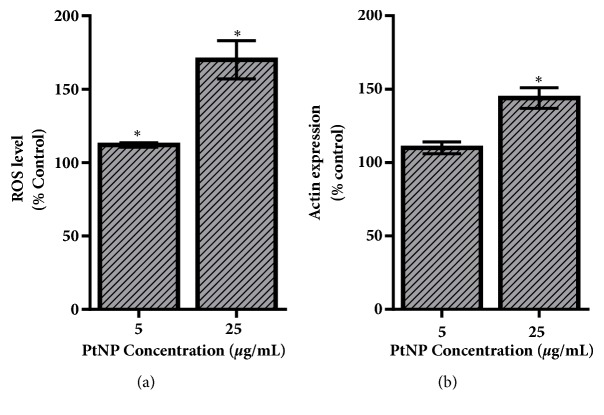
PtNP-induced stress response within the HepG2 cell model. (a) ROS and (b) actin levels were measured within HepG2 cells following a 24-hour exposure to varying dosages of PtNPs. Both endpoints demonstrated a dose-dependent increase in stress activation. Data represents 3 independent trials with *∗* denoting statistical significance from untreated controls (*p* < 0.05).

One of the areas of conflicting reports following PtNP exposure is the activation of intracellular stress, with studies identifying both pro- and anti-oxidant effects [14–17]. Therefore, the next goal was to characterize the HepG2 stress response following exposure to the experimental, 70 nm PtNPs (Figure 3). First, intracellular ROS levels were monitored, as its production is a documented precursor for apoptosis and a known cellular response following NP exposure [26, 27]. As shown in Figure 3(a), the PtNPs induced ROS production in a dose-dependent fashion, with a substantial response associated with the 25 *μ*g/mL condition.

In addition to ROS, actin expression was evaluated as a metric for cellular stress. Actin becomes disorganized and inflamed during stress, making an increase in its expression directly proportional to cellular distress [28]. Following PtNP exposure, the actin expression displayed a dose-dependent increase, closely mirroring the ROS results (Figure 3(b)). Taken together, these findings demonstrated that citrate coated, 70 nm PtNPs were able to activate a significant stress response in HepG2 liver cells, even in the absence of cytotoxicity.

### 3.3. Inflammatory Response to PtNP Exposure

Beyond activation of stress, NP exposure has been shown to trigger inflammatory and immune responses in mammalian cells [5, 29]. Assessing inflammatory activation is not a traditional nanotoxicological outcome; however, sustained inflammation can introduce serious health implications, including heart disease, hypertension, and even cancer [30]. To assess PtNP-induced inflammation in HepG2 cells, the secreted levels of IL-1*β*, IL-6, IL-8, and TNF-*α* were measured, which are early markers of an active inflammatory response [31].

**Figure 4:**
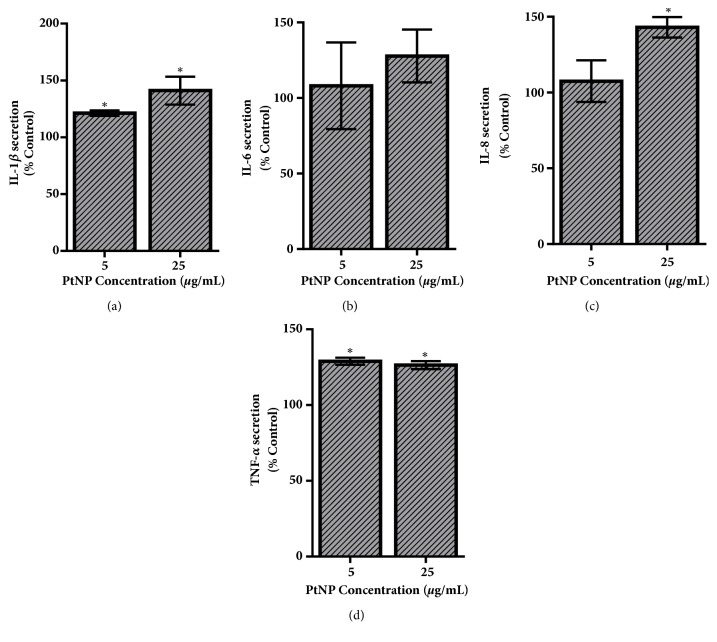
Activation of the HepG2 inflammatory response. To assess PtNP-dependent inflammatory activation, secretion levels of key proinflammatory cytokines were quantified. Following a 24-hour exposure to varying levels of PtNPs, the media were recovered from the HepG2 culture and underwent analysis for (a) IL-1*β*, (b) IL-6, (c) IL-8, and (d) TNF-*α*. Data represents 3 independent trials with *∗* denoting statistical significance from untreated controls (*p* < 0.05).

The cytokine production levels following a 24-hour exposure to PtNPs are provided in Figure 4. Secretion of both IL-1*β* and IL-8 increased, in a dose-dependent manner, with the 25 *μ*g/mL dosage raising production approximately 50% over the untreated control. PtNP upregulated TNF-*α* formation, approximately 25%, but appeared to be independent of exposure concentration. This study identified no significant changes to IL-6 levels following PtNP exposure.

### 3.4. Modified IGF-1 Signaling

Lastly the ability of PtNPs to disrupt signal transduction was explored, as nanomaterials have previously been shown to modulate signaling pathways following growth factor stimulation [7, 17]. Signal transduction is a foundational aspect of cellular functionality as its activation and regulation control numerous outcomes including proliferation, migration, and survival. Moreover, unregulated signaling has been correlated to severe health concerns including cancer, respiratory conditions, and neurological diseases [32].

**Figure 5:**
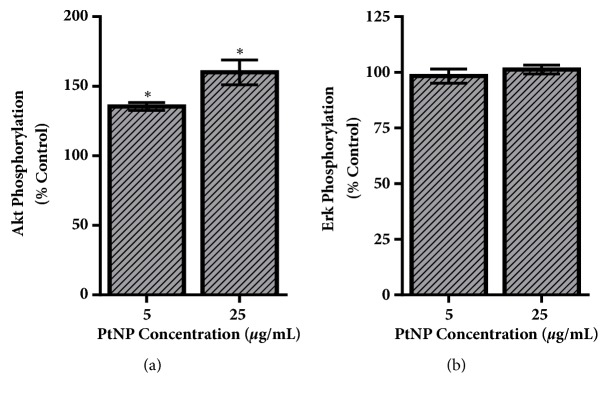
Evaluation of IGF-1 dependent signal transduction. HepG2 cells were exposed to PtNPs and then stimulated with IGF-1 to induce the PI3K/Akt and Ras/Erk signaling pathways. Activation of these cascades was assessed via normalized phosphorylation levels of (a) Akt and (b) Erk, respectively. Data represents 3 independent trials with *∗* denoting statistical significance from untreated controls (*p* < 0.05).

IGF-1 is a known growth factor for HepG2 cells, with ligand-receptor binding inducing the critical signaling pathways of PI3K/Akt and Ras/Erk [33]. Activation of these pathways was quantified by evaluating phosphorylation levels of Akt and Erk, respectively (Figure 5), which are recognized central players in cellular functionality. As seen in Figure 5(a), both experimental PtNP dosages augmented Akt activation, in a dose-dependent fashion. Following 25 *μ*g/mL PtNP exposure, Akt phosphorylation was increased by approximately 50% over untreated controls. On the contrary, Erk phosphorylation levels were the same for all exposure conditions, suggesting that IGF-1 dependent activation of the Ras/Erk cascade was not a PtNP cellular target in HepG2 cells.

### 3.5. Implications of These Findings

**Figure 6:**
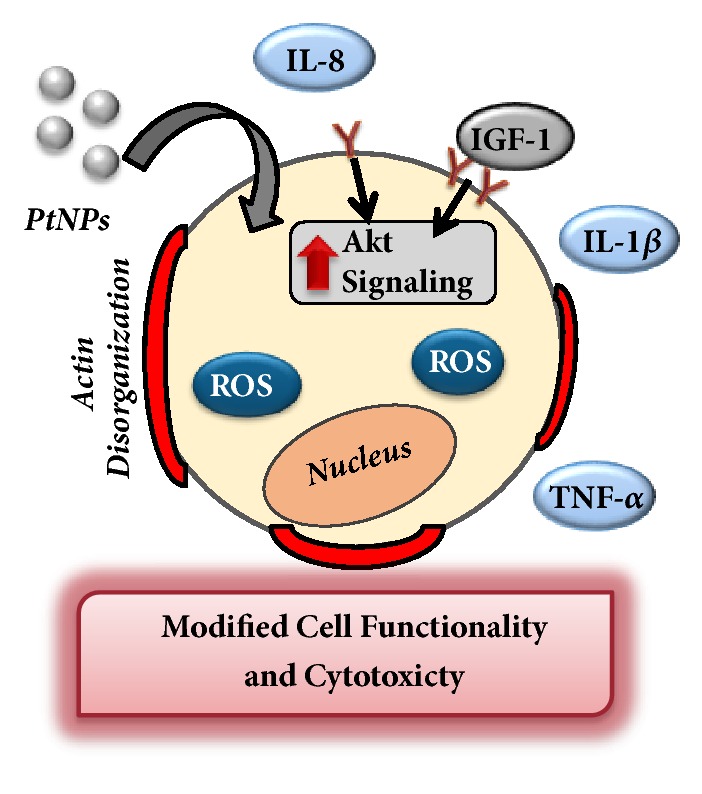
Summary of HepG2 cellular responses following PtNP exposure. This study identified that PtNPs induced traditional toxicological responses including cytotoxicity and stress activation. Moreover, cellular functionality was modified as assessed via activation of an inflammatory response and modulation of IGF-1 dependent signal transduction.

The goal of this study was to further elucidate the biological response of liver cells following exposure to PtNPs. To date the literature has produced conflicting reports regarding PtNP safety [13–16]. The most likely explanation is that nanotoxicological outcomes are dependent upon both cell type and the unique physicochemical properties of the experimental particles. This work utilized a human liver model, HepG2, as all NPs, including PtNPs, are known to accumulate in the liver [13]. Characterization assessments demonstrated that the citrate coated 70 nm PtNPs were of high quality, uniform in size, and stable within solution (Figure 1 and Table 1). The work presented here focused on two distinct areas: (1) traditional toxicological endpoints and (2) evaluation of cellular functionality in the absence of toxicity. A pictorial summary of all observed HepG2 responses following PtNP exposure is shown in Figure 6.

Initial efforts focused on traditional toxicological endpoints, with the first evaluation metric exploring PtNP-induced cytotoxicity of HepG2 cells (Figure 2). A dose-dependent toxicity was identified, with a 25% loss of viability only occurring at the highest experimental PtNP concentration, 100 *μ*g/mL. While this observed HepG2 cytotoxicity is significant, PtNPs are not renowned for their toxicity potential versus some other core compositions, such as silver. In a recent study, 10 nm silver NPs were correlated with an approximate 60% loss of human lung cell viability following a 24-hour exposure to 25 *μ*g/mL concentration [7]. A previous study compared the direct toxicity of several NPs and identified that silver, copper, and zinc were some of the most potent NP cores, inducing exceptionally high degrees of cytotoxicity [34], helping to highlight that PtNPs can cause cell death, but not to the same degree as other experimental nanomaterials.

However, at lower, application-relevant dosages, full viability was maintained, suggesting that a direct toxicity response would not transpire if PtNPs were incorporated into products and therapeutics. However, even in the absence of cytotoxicity, PtNPs were found to induce intracellular stress pathways including ROS (Figure 3). ROS is a prooxidant cellular response to NPs and is a well-documented precursor of additional cellular distress, activation of inflammatory and immune responses, and genetic modifications [27, 35]. One known downstream response of ROS production is actin inflammation and disorganization [28]. This response was verified following PtNP exposure, with a 50% augmentation of actin expression identified. Therefore, this study confirmed that at low, subtoxic concentrations, PtNPs were still capable of inducing multiple stress responses.

Of greater interest was the investigation into whether PtNP exposure could disrupt normal HepG2 functionality. In addition to being a nontraditional toxicity evaluation, the examination of inflammatory responses and signaling activation would provide insight into whether PtNPs could alter basal cell function and activity. Following PtNP exposure, secretion of IL-1*β*, IL-8, and TNF-*α* was all upregulated (Figure 4), indicating an active inflammatory response. Cytokine production is a defence mechanism that is triggered following extensive cellular stress [30, 31] and therefore aligns with the ROS results. Moreover, this study identified that PtNP incubation interfered with the HepG2 response to IGF-1 stimulation, increasing Akt phosphorylation levels. This result aligns with a previous report which identified that 5.8 nm PtNPs increased activation of multiple signaling pathways, including PI3K/Akt, Ras/Erk, and Ras/JNK, in keratinocytes [17]. This work, which examined the effect of 70 nm PtNPs on a liver model, did not uncover any changes to the Ras/Erk cascade, further supporting the supposition that PtNP-dependent biological responses are reliant upon both cell type and NP physicochemical properties. Taken together, this work identified that PtNP exposure augmented both inflammation and signal transduction within HepG2 cells. Combined these responses could lead to serious health concerns if chronic exposure occurred, as alterations to these functional processes have been correlated to numerous diseases, including cancer, heart disease, and neurological disorders [30, 32, 35]. Moreover, increased Akt phosphorylation can trigger a deeper inflammatory response [36], thereby establishing a synergistic intracellular reaction.

## 4. Conclusions

This study explored the safety of 70 nm, citrate coated PtNPs within a liver mammalian model. The PtNPs induced a potent cytotoxic response only at high dosages, well above incidental exposure levels. However, at lower concentrations, even in the absence of cellular death, PtNP exposure elicited a significant stress response, notably a marked increase in ROS production. Aligning with the stress activation, a strong inflammatory reaction was observed, through both actin inflammation and augmented secretion of IL-1*β*, IL-8, and TNF-*α*. Following exposure, it was elucidated that PtNPs disrupted basal cellular functionality as assessed via modulation to the PI3K/Akt signaling pathway following IGF-1 stimulation. Therefore, while low level exposure to PtNPs may not induce direct toxicity, the combined presence of intracellular stress, active inflammatory responses, and upregulated signal transduction have the potential to introduce long term health hazards. However, as PtNP-dependent biological responses are correlated to cell type and physicochemical parameters, further investigations are required prior to their regulated utilization in consumer applications or as biomedical therapeutics.

## Figures and Tables

**Table 1:** PtNP characterization assessments.

**Primary size (nm)**	**Agglomerate size (nm)**		**PdI**		**Zeta potential (mV)**	
	*Water*	*Media*	*Water*	*Media*	*Water*	*Media*
68.3 ± 3.5	85.9 ± 3.5	112.4 ± 7.0	0.158	0.248	-40.6 ± 1.1	-12.2 ± 0.9

## Data Availability

The data used to support the findings of this study are included within the article.
